# Monoterpenes Differently Regulate Acid-Sensitive and Mechano-Gated K_2P_ Channels

**DOI:** 10.3389/fphar.2020.00704

**Published:** 2020-05-20

**Authors:** Eden Arazi, Galit Blecher, Noam Zilberberg

**Affiliations:** ^1^ Department of Life Sciences Ben-Gurion University of the Negev, Beer-Sheva, Israel; ^2^ The Zlotowski Center for Neuroscience, Ben-Gurion University of the Negev, Beer-Sheva, Israel

**Keywords:** K_2P_ channel, TREK-1, TRAAK, TASK-1, TALK, monoterpenes, leak channels, voltage-dependent current

## Abstract

Potassium K_2P_ (“leak”) channels conduct current across the entire physiological voltage range and carry leak or “background” currents that are, in part, time- and voltage-independent. The activity of K_2P_ channels affects numerous physiological processes, such as cardiac function, pain perception, depression, neuroprotection, and cancer development. We have recently established that, when expressed in *Xenopus laevis* oocytes, K_2P_2.1 (TREK-1) channels are activated by several monoterpenes (MTs). Here, we show that, within a few minutes of exposure, other mechano-gated K_2P_ channels, K_2P_4.1 (TRAAK) and K_2P_10.1 (TREK-2), are opened by monoterpenes as well (up to an eightfold increase in current). Furthermor\e, carvacrol and cinnamaldehyde robustly enhance currents of the alkaline-sensitive K_2P_5.1 (up to a 17-fold increase in current). Other members of the K_2P_ potassium channels, K_2P_17.1, K_2P_18.1, but not K_2P_16.1, were also activated by various MTs. Conversely, the activity of members of the acid-sensitive (TASK) K_2P_ channels (K_2P_3.1 and K_2P_9.1) was rapidly decreased by monoterpenes. We found that MT selectively decreased the voltage-dependent portion of the current and that current inhibition was reduced with the elevation of external K^+^ concentration. These findings suggest that penetration of MTs into the outer leaflet of the membrane results in immediate changes at the selectivity filter of members of the TASK channel family. Thus, we suggest MTs as promising new tools for the study of K_2P_ channels’ activity *in vitro* as well as *in vivo*.

## Introduction

Potassium channels selectively and rapidly enable the movement of K^+^ ions across biological membranes down the electrochemical K^+^ gradient at a rate close to that of diffusion (Mackinnon, 2003). Members of the potassium leak channel family are structurally unique among potassium channels since each subunit possesses four transmembrane segments and two pore-forming domains (2P/4TM architecture). As such, these channels are often referred to as two pore-domain K^+^ or K_2P_ channels (Goldstein et al., 2001; Choe, 2002). These channels conduct current across the entire physiological voltage range and are essential for neurophysiological function, while their activity modulates excitability. It was shown that K_2P_ channels could also increase excitability by supporting high-frequency firing once an action potential threshold is reached (Brickley et al., 2007). It was recently reported that the majority of K_2P_ channels are gated by membrane potential in spite of their lack of a voltage sensor, as the outward current of K^+^ ions through the selectivity filter was found to open this gate (Schewe et al., 2016). Members of this family may react to membrane stretch, as well as to intracellular and extracellular pH changes, phosphorylation, the activity of various G-protein coupled receptors, and more (Goldstein et al., 2001; Brickley et al., 2007; Demeure et al., 2011; Feliciangeli et al., 2015). K_2P_ channels activity was shown to modulate various important physiological processes such as pain perception (Alloui et al., 2006) and cardiac activity (Ellinghaus et al., 2005; Decher et al., 2017; Schmidt et al., 2017). Human K_2P_3.1 channels (TASK-1) are expressed mainly in the atria and possess a promising target for atrial fibrillation treatment (Schmidt et al., 2014; Schmidt et al., 2018). A mutation in K_2P_9.1 (TASK-3) is connected to the Birk–Barel syndrome, mental retardation, and unique dysmorphism syndrome (Barel et al., 2008). Also, the effect of several volatile analgesics is mediated, in part, through their action on K_2P_2.1 and K_2P_4.1 (TRAAK) (Franks and Honore, 2004).

Terpenes are a large group of structurally diverse organic chemicals that are mostly produced in plants. Monoterpenes (MTs) are terpenes that are composed of two five-carbon isoprene units. For centuries, MTs have been known for their beneficial effects as antifungal agents (Marei et al., 2012), antibacterial (Garcia et al., 2008), and analgesic (Khalilzadeh et al., 2016) agents. Terpenes have been proposed as remedies for the treatment of pain (Quintans-Junior et al., 2013; Quintans Jde et al., 2013; Guimaraes et al., 2014) and cardiovascular diseases (Magyar et al., 2004; Aydin et al., 2007; Menezes et al., 2010; Peixoto-Neves et al., 2010; Santos et al., 2011), and were shown to possess antitumor, local anesthetic, and anti-ischemic abilities (Koziol et al., 2014).

Several MTs were found to affect ion channels, both in excitable cells (Oz et al., 2015) and in other tissues (Muruganathan et al., 2017). To name a few, carvacrol and thymol were found to activate and sensitize the murine and human transient receptor potential (TRP) channel TRPV3, and acyclic MTs like citronellol, nerol, and their derivatives were found to modulate the activity of TRPA1 (Ortar et al., 2014). MTs were found to act upon other TRP channels (Xu et al., 2006; Parnas et al., 2009), as well as on voltage-gated ion channels and GABA receptors (Czyzewska and Mozrzymas, 2013; Kawasaki et al., 2013). However, their activity on members of the K_2P_ potassium channels had not yet been studied.

Recently (Arazi et al., 2020), we reported the activation of K_2P_2.1 by various MTs. Here, we report that MTs activate the other two mechano-gated K_2P_ channels (*i.e.*, K_2P_4.1 and K_2P_10.1), in addition to members of other groups of K_2P_ channel families (*e.g.*, TALK, TRESK). Moreover, we found that MTs display remarkable selectivity towards the different K_2P_ channels, and we report that they selectively inhibited the voltage-dependent current of TASK family members.

## Methods

### Animals

All experiments using animals were performed following the guidelines of the Institutional Animal Care and Use Committee. The project approval number is IL-61-09-2015.

### Cloning

Channels were cloned into plasmid pRAT that included a T7 RNA polymerase promoter to enable cRNA synthesis, as well as the 3′-UTR and 5′-UTR sequences of the *Xenopus laevis* β-actin gene to ensure efficient expression in *Xenopus* oocytes. Competent *Escherichia coli* DH5*α* cells were transformed by heat shock. Plasmid DNA was purified with a Wizard Plus SV Miniprep kit (Promega). Restriction enzyme digestions were performed according to the manufacturer’s instructions (Fermentas or NEB). Point mutations were generated according to the Quickchange site-directed mutagenesis technique (Stratagene) and confirmed by sequencing. cRNA was transcribed *in vitro* by T7 polymerase using an AmpliCap High Yield Message Maker (Epicentre) kit.

### Electrophysiology

*Xenopus laevis* oocytes were isolated and injected with 20–40 nl of solutions containing 0.3–40 ng cRNA using a 3.5″ Drammond#3-000-203-G/X glass capillary, pulled in a Sutter P97 capillary puller and a Drummond manual oocyte microinjection pipette (3-000-510-X). Whole-cell currents were measured 1–3 days after injection by the two-electrode voltage-clamp technique (GeneClamp 500B, Axon Instruments). Data were filtered at 2 kHz and sampled at 5 kHz with Clampex 9.0 software (Axon Instruments). For two-electrode voltage-clamp experiments, the pipette contained 3M KCl and the bath solution contained (in mM) unless otherwise noted: 4 KCl, 96 NaCl, 1 MgCl_2_, 0.3 CaCl_2_, 5 HEPES, pH 7.4 with NaOH (standard solution). All measurements of K_2P_5.1 and K_2P_17.1 channels were performed at pH = 9.0. When needed, bath solution sodium ions were isotonically replaced by potassium ions and *vice versa*. When testing MT activity, the standard bath solution was supplemented with the same concentration of the solvent (ethanol) as of the tested chemical.

Injection of cRNA into oocytes was done in OR-2 solution (in mM: 5 HEPES, 1 MgCl_2_, 2.5 KCl, 82.5 NaCl, pH = 7.4). Post-injection oocytes were maintained in ND-91 solution (in mM: 5 HEPES, 1 MgCl_2_, 1.8 CaCl_2_, 2 KCl, 91 NaCl, pH = 7.4). Specific recording protocols are mentioned in the relevant figure legends. To determine the voltage-dependent fraction of the current, the initial, voltage-independent (instantaneous) current was estimated by fitting the current to an exponential decay slope as the initial currents are masked by the capacitive transient current, as was previously described (Arazi et al., 2020).

### Chemicals

Carvacrol (cat#282197), thymol (cat#T0501), p-cymene (cat#C121452), 4-isopropylphenol (cat# 175404), eugenol (cat#E51791, cinnamaldehyde (cat#W228613), menthol (cat#M2772), beta-citronelol (cat# C83201), geraniol (cat#16333, 4-methylcatechole (cat# M34200), and arachidonic acid (cat#A3611) were all purchased from Sigma-Aldrich.

### Preparation of Compounds

Compounds delivered as powders were dissolved into stock solutions (4–6 M) in 100% ethanol. Compounds delivered as liquid oils (6.5–7.5 M) were diluted 1:1 with ethanol to form stock solutions and were kept at −20°C for up to two weeks. Just before testing, stock solutions were diluted in the bath solution to the desired concentration, and diluted compounds were vigorously vortexed until completely dissolved. All solutions were supplemented with ethanol to a final concentration of 0.1% (v/v) (confirmed not to harm the oocytes). The pH was corrected to 7.4 ± 0.05 using NaOH or HCl.

### Statistical Analysis

Data were expressed as the mean ± standard error of the mean (SEM) and analyzed and presented using Microsoft Excel 2016. Groups of two paired data sets were analyzed using a Wilcoxon Signed Ranks test and groups of two unpaired data sets were analyzed using Mann–Whitney U test with IBM SPSS Statistics ver. 20 software. Values were considered to be significantly different when the z-value was ≤0.05 (*), ≤0.01 (**), or ≤0.001 (***). All experiments were repeated with at least five oocytes.

## Results

### Activation of Mechano-Gated Channels by Monoterpenes

**Figure 1:**
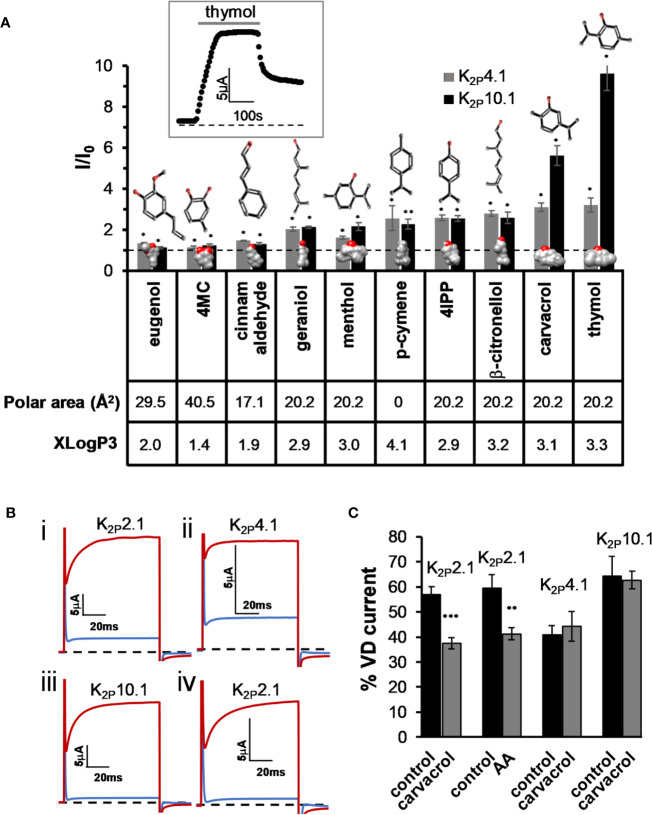
Activation of K_2P_4.1 and K_2P_10.1 by monoterpenes. **(A)** Activation of K_2P_4.1 and K_2P_10.1. Oocyte membrane potential was held at −80 mV and pulsed to +25 mV for 75 ms with 5 s interpulse intervals. All MTs were applied at the same concentration (0.3 mM), and currents were measured after 4 min (mean ± S.E., n = 5–10). Polar area (Å^2^) and octanol–water partition coefficient (logP) prediction (XLogP3) were obtained from PubChem (Kim et al., 2016). 2D structures and the coordinates for the 3D structures of the terpenes were obtained from ChemSpider. 3D models were performed with the UCSF Chimera package (Pettersen et al., 2004). Oxygen molecules are colored red. The dashed line represents no change from the initial current. Inset- currents of a representative oocyte expressing K_2P_10.1 before, during and after thymol application. **(B)** Currents at 60 mV before (in red) and during (in blue) application of carvacrol (i–iii) or arachidonic acid (AA) (iv), on K_2P_2,1 (i, iv), K_2P_4.1 (ii), and K_2P_10.1 (iii) **(C)**. Fraction of voltage-dependent current (in %) before (black) and after (gray) application of 0.3 mM carvacrol or arachidonic acid (AA, 100 µM). A fit of the current (at 60 mV) to an exponential decay slope was used to identify the initial current (mean ± S.E., n = 6–9).

As we have recently reported (Arazi et al., 2020), the activity of K_2P_2.1 is modulated by various MTs. K_2P_2.1 is a member of the mechano-gated K_2P_ channel clade that includes K_2P_4.1 (TRAAK) and K_2P_10.1 (TREK-2). We, therefore, investigated whether MTs modulate all mechano-gated K_2P_ channels. An external application of MTs resulted in the increase in currents of K_2P_10.1 channels by seven compounds and in the increase in currents of K_2P_4.1 channels by eight of the tested compounds, although to lower levels (**Figure 1A**). As was found for K_2P_2.1 (Arazi et al., 2020), the phenol-containing compounds (carvacrol and thymol) were more potent in opening both channels, while linear compounds and compounds containing no hydroxyl group were less effective (**Figure 1A**). Under standard testing conditions, currents of most K_2P_ channels are composed of two components: an instantaneous “leak” current (voltage-independent, VI) and a voltage-dependent (VD) current (Schewe et al., 2016), as demonstrated in **Figure 1B**. We, thus, looked at whether MTs affect the voltage sensitivity of the channels by looking at the change in the proportion of the two current components. For K_2P_4.1 and K_2P_10.1 channels, no change in voltage dependency was detected (**Figure 1C**). However, for K_2P_2.1 channels, voltage dependency was reduced during carvacrol application, as was previously reported (Arazi et al., 2020). As expected (Schewe et al., 2016), arachidonic acid had a similar effect to carvacrol (**Figure 1C**).

### Activation of TALK and TRESK Channels

**Figure 2:**
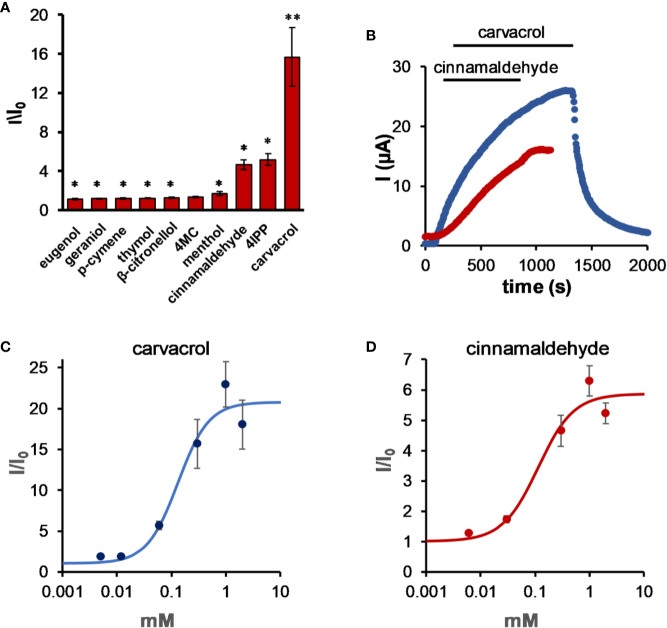
Carvacrol and cinnamaldehyde robustly activate K_2P_5.1. **(A)** Activation of K_2P_5.1 by monoterpenes. Oocyte membrane potential was held at −80mV and pulsed to +25 mV for 75 ms with 5 s interpulse intervals. All MTs were applied at the same concentration (0.3 mM), and currents were measured after 5 min of incubation (mean ± S.E., n = 6–10). **(B)** Time course for activation by 0.3 mM carvacrol and 0.3 mM cinnamaldehyde for representative oocytes expressing K_2P_5.1 channels. Currents were measured as in **(A)**. **(C)** Carvacrol dose-response for K_2P_5.1 channels (mean ± S.E., n = 5–8) (EC_50_ = 0.13 ± 0.05 mM). **(D)** Cinnamaldehyde dose–response for K_2P_5.1 channels (mean ± S.E., n = 5–8) (EC_50_ = 0.11 ± 0.07 mM). Currents were measured at 25 mV, as in **(A)**, after incubation for 5 min. *p ≤ 0.05, **p ≤ 0.01.

The activity of members of the TALK clade of potassium channels (K_2P_5.1, K_2P_16.1, and K_2P_17.1; TASK-2, TALK-1, and TALK-2, respectively) is sensitive to external pH, as these channels are activated at an alkaline pH (Decher et al., 2001; Girard et al., 2001). K_2P_5.1 (TASK-2) is expressed mostly at the tubular epithelial and is involved in pathological conditions such as Balkan endemic nephropathy (BEN) (Toncheva et al., 2014; Reed et al., 2016). While most tested compounds had almost no effect on this channel, carvacrol and cinnamaldehyde (**Figure 2**) activated it by up to 17-fold (15.7 ± 3.0, n = 7 and 4.7 ± 0.5, n = 5, respectively, 0.3 mM for both compounds), although at different rates (**Figure 2B**). While activation by carvacrol was reversible, activation by cinnamaldehyde was not, even after a 5-min wash. It should be noted that irreversible activation of TRPA1 channels by cinnamaldehyde was previously reported (Macpherson et al., 2007). The minimal concentration that showed substantial activation of the channel was 12 µM for carvacrol (albeit not statistically significant) (1.9 ± 0.1-fold, n = 5, **Figure 2C**) and 30 µM for cinnamaldehyde (2.8 ± 0.3-fold, n = 5; statistically significant).

**Figure 3:**
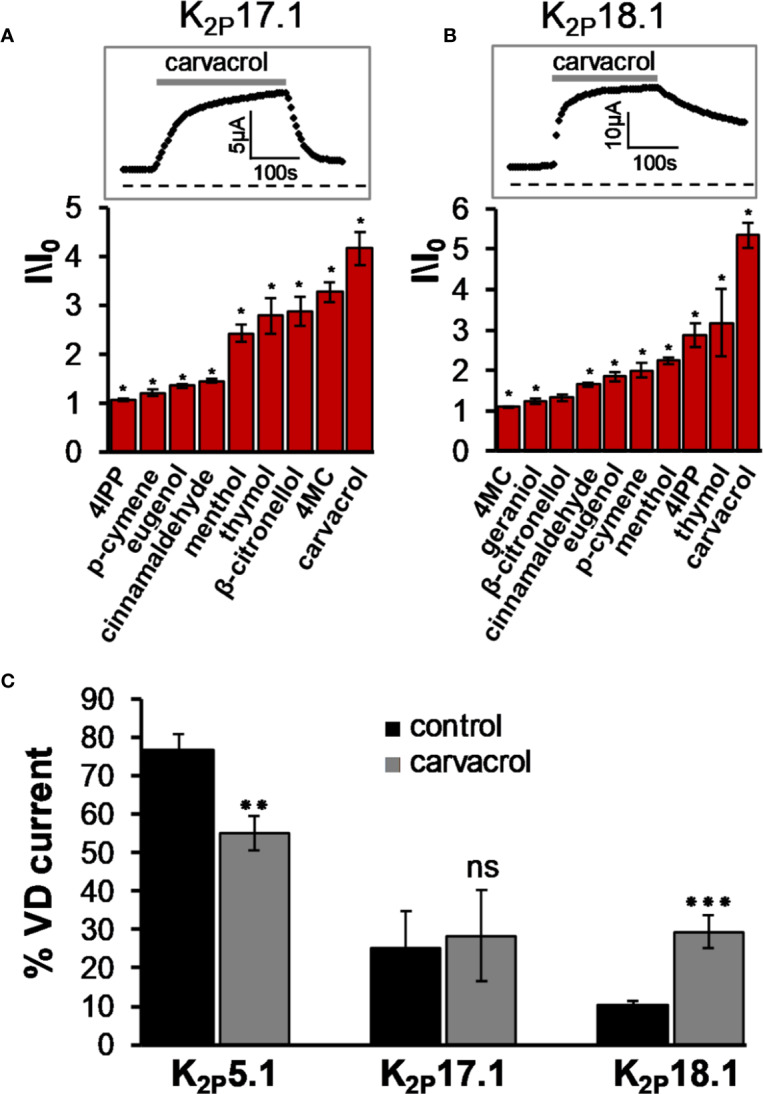
K_2P_17.1 and K_2P_18.1 are activated by monoterpenes. **(A)**, **(B)** Activation of K_2P_17.1 **(A)** and K_2P_18.1 **(B)** by monoterpenes. Oocyte membrane potential was held at −80 mV and pulsed to +25 mV for 75 ms with 5 s interpulse intervals. All MTs were applied at the concentration of 0.3 mM, and currents were measured 4 min after application of the indicated monoterpene (mean ± S.E., n = 5–10). Insets: currents of representative oocytes expressing K_2P_17.1 **(A)** and K_2P_18.1 **(B)** during application of 0.3 mM carvacrol. **(C)** Fraction of voltage-dependent (VD) current (in %) under control conditions and after application of 0.3 mM carvacrol for three channel types, as indicated. Oocytes were held at −80mV, and currents were measured at 30 mV. A fit of the results to an exponential decay slope was used to identify the initial current (mean ± S.E., n = 6–9). *p ≤ 0.05, **p ≤ 0.01, ***p ≤ 0.001, ns, not significant.

K_2P_16.1 and K_2P_17.1 are expressed predominantly in the pancreas and may be involved in the exocrine secretion of bicarbonate. A gain of function mutation in K_2P_17.1 was associated with progressive cardiac conduction disorder (Friedrich et al., 2014). While K_2P_16.1 was not affected by any of the tested MTs (not shown), K_2P_17.1 was moderately activated by menthol, thymol, *β*-citronellol, 4MC, and carvacrol (**Figure 3A**). K_2P_18.1 (TRESK, KCNK18) is unique among other K_2P_ channels by having an extra-long cytoplasmatic domain that is located between the two pore-forming domains (Sano et al., 2003). This channel is expressed in the dorsal root ganglion, trigeminal ganglion neurons, and spinal cord (Sano et al., 2003; Kang and Kim, 2006; Dobler et al., 2007). Mutation in this channel was linked to familial migraine with aurora (Lafreniere et al., 2010). K_2P_18.1 was opened rapidly and robustly by carvacrol and to a lesser degree by thymol and 4-isopropylphenol (4IPP) (**Figure 3B**). Other compounds displayed mild to no effect on this channel. Since with mechano-gated K_2P_ channels, we observed a reduction in the proportion of the voltage-dependent current as a result of activation by carvacrol (**Figure 1C**), we tested this feature in these channels as well. In K_2P_5.1 channels, the share of the voltage-dependent current indeed decreased (**Figure 3C**). In contrast, for K_2P_17.1 channels, the share of the voltage-dependent current did not change. In K_2P_18.1 channels, where the basal share of the voltage-dependent current was low, currents displayed more sensitivity to voltage after incubation with carvacrol (**Figure 3C**).

### Acid-Sensitive K_2P_3.1 and K_2P_9.1 (TASK) Channels Are Inhibited by Monoterpenes

**Figure 4:**
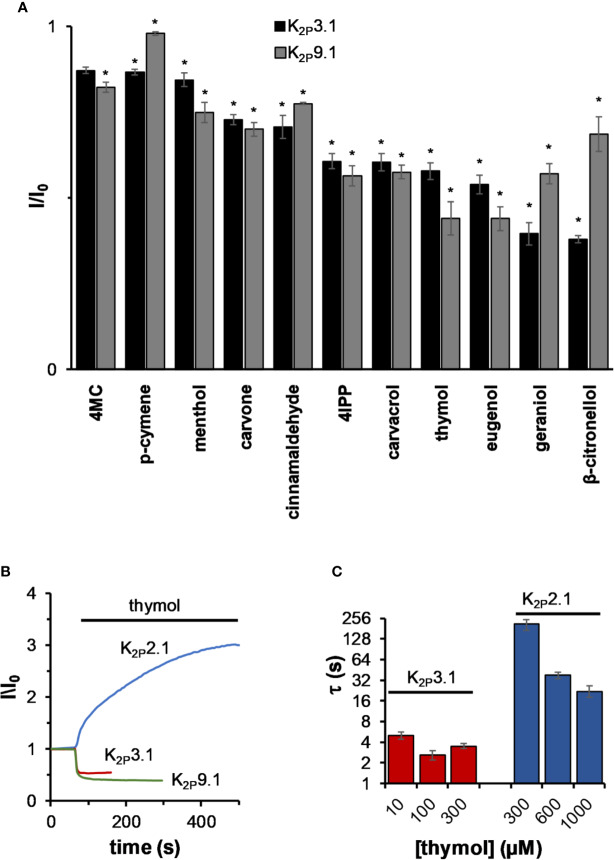
The effect of monoterpenes on acid-sensitive K_2P_ channels. **(A)** Inhibition of K_2P_3,1 and K_2P_9.1 currents. Currents were measured before and after 2 in incubation with the indicated MT (mean ± S.E., n = 5–10). All MTs were applied at a concentration of 0.3 mM. **(B)** Normalized currents during 0.3 mM thymol application for representative oocytes expressing either K_2P_2.1, K_2P_3.1, and K_2P_9.1. **(C)** The time constant (τ) of current changes during the application of thymol at different concentrations for K_2P_2.1 or K_2P_3.1 (mean ± S.E., n = 6–10). *p ≤ 0.05.

K_2P_3.1 and K_2P_9.1 (TASK-1 and TASK-3, respectively) channels are expressed in the pancreas and placenta and to a lesser degree in the brain, heart, and kidneys (Duprat et al., 1997; Kim et al., 2000). Unlike all other tested K_2P_ channels, current levels of K_2P_3.1 and K_2P_9.1 decreased by all tested MTs (**Figure 4A**). In contrast to mechano-gated channels, which were affected mostly by cyclic phenolic compounds like thymol and carvacrol, TASK channels were affected mostly by linear MTs such as *β*-citronellol and geraniol (**Figure 4A**). Inhibition was rapid (*e.g.* τ = 3.8 ± 0.2, 6.2 ± 1.2, and 5.6 ± 1.5 s for thymol, *β*-citronellol, and carvacrol, respectively, n = 7–11) and within the solution change rate in our system (τ = 7.7 ± 1.0 s, n = 5). When using thymol, a monoterpene that affects both channel types, it was obvious that the inhibition rate was remarkably faster than that observed for activation of mechano-gated K_2P_ channels (**Figure 4B**). To make sure that the differences in rates were not merely a result of a difference in affinities (K_inhibition_ of K_2P_3.1 by thymol is 20 ± 5 µM and K_activation_ of K_2P_2.1 by thymol is 290 ± 50 µM, not shown), we measured the current change rates at three different concentrations for each channel (**Figure 4C**). As expected, no measurable change in the rate of K_2P_3.1 channels inhibition was observed, while the activation rate for K_2P_2.1 channels was much slower at all concentrations.

**Figure 5:**
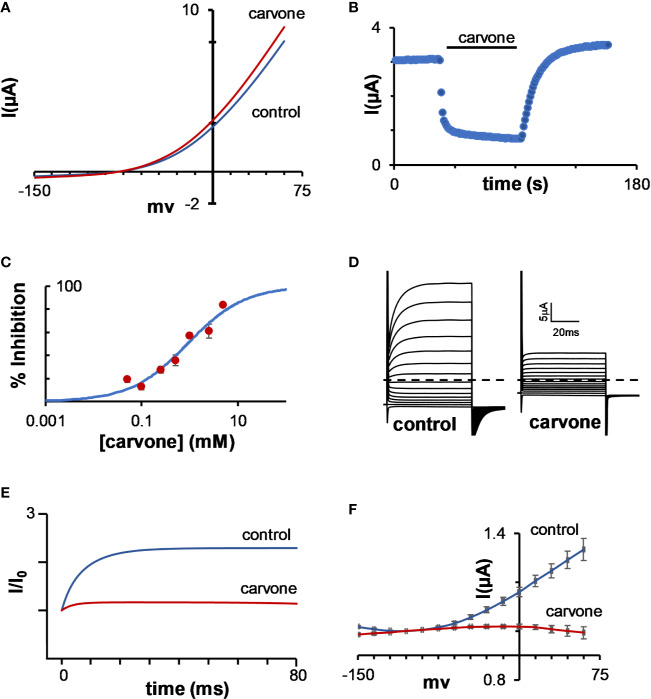
Inhibition of K_2P_3.1 by carvone. **(A)** Current–voltage relationship of a representative oocyte expressing K_2P_2.1, before and after application of 1mM carvone. **(B)** The current of a representative oocyte expressing K_2P_3.1 during incubation with 1 mM carvone. Oocyte membrane potential was held at −80 mV and pulsed to +25 mV for 75 ms with 1 s interpulse intervals. **(C)** Carvone dose–response for K_2P_3.1 channels (mean ± S.E., n = 6–10) (K_inhibition_ = 0.90 ± 0.16 mM). **(D)** Currents of a representative oocyte expressing K_2P_3.1 channels before and during incubation with 1 mM carvone at 20 mM potassium at the bath. The oocyte was held at −80 mV, then at −135 mV for 30 ms, and then pulsed from −150 mV to 60 mV in 15 mV intervals. The dashed line represents zero current. **(E)** Currents at 60 mV of a representative oocyte before and during incubation with 1 mM carvone. Currents were normalized to the initial current. A fit of the results to an exponential decay slope was used to identify the initial current. **(F)** Tail analysis of currents before and during incubation with 1 mM carvone at external potassium concentration of 100 mM (mean ± S.E., n = 6). For each oocyte, currents were normalized to the current at −105 mV.

To further examine the unique activity of MTs on TASK channels, we tested the activity of carvone on K_2P_3.1 channels as a model, as this monoterpene had no activity on K_2P_2.1 channels (**Figure 5A**), while readily and rapidly (**Figure 5B**) decreasing K_2P_3.1 channel currents with a K_inhibition_ of 0.90 ± 0.16 mM (**Figure 5C**). As in most K_2P_ channels, when held at −80 mV, K_2P_3.1 currents are comprised of two components: an instantaneous, voltage-independent (VI), and a time- and voltage-dependent component (VD) (**Figure 5D**, control). As was observed with other MTs, the VD component of the current was dramatically reduced due to application of carvone (**Figures 5D, E**). The inhibition of the VD currents resulted, as anticipated, in the disappearance of K_2P_3.1 channels tail currents (**Figure 5F**).

**Figure 6:**
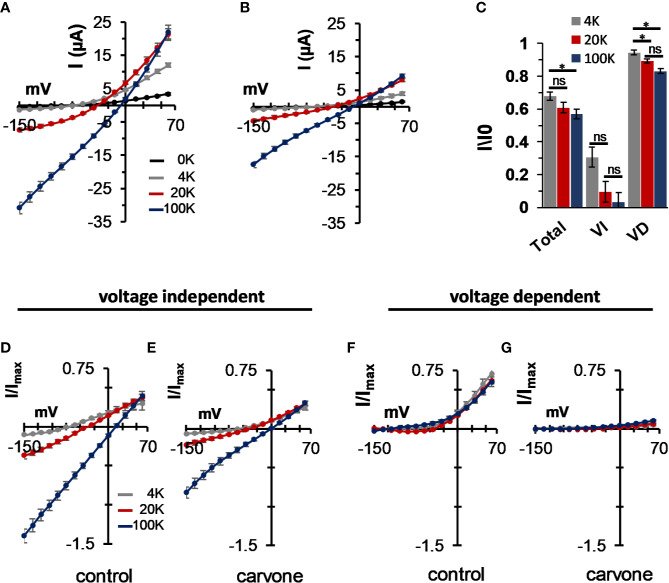
The effect of external K^+^ on the inhibition of the voltage-dependent current in K_2P_3.1. **(A, B)** Steady-state current–voltage relationships for oocytes expressing K_2P_3.1 at four external potassium concentrations (0, 4, 20, and 100 mM) under control conditions **(A)** or after incubation with 1 mM carvone **(B)**. Oocytes were held at −80 mV, pulsed to −135 mV for 30 ms, and then pulsed from −150 mV to 60 mV in 15 mV voltage intervals (mean ± S.E., n = 6–9). **(C)** The fraction of inhibited current due to carvone application of the total current (Total) and its components: the voltage-independent (VI) and the voltage-dependent (VD) currents. Currents at 60 mV were tested at three external potassium concentrations (4, 20, and 100 mM) (mean ± S.E., n = 6–9). **(D–G)** Current–voltage relationships for oocytes expressing K_2P_3.1 channels at three different external potassium concentrations, as indicated (mean ± S.E., n = 6–9). Currents were measured as in **(A)**. The voltage-independent **(D, E)** and the voltage-dependent **(F, G)** fractions of the current were calculated as in **Figure 1C** and are presented individually. Measurements were performed before **(D, F)** and after **(E, G)** application of 1 mM carvone. *p ≤ 0.05, ns, not significant.

External K^+^ concentration is known to affect the open probability of the selectivity filter gate of potassium channels (Hille, 2001) and, in particular, that of K_2P_ channels (Zilberberg et al., 2001). We, thus, tested the effect of external K^+^ on carvone-induced current inhibition. The concentration of external K^+^ had a profound effect on K_2P_3.1 currents. A significant current decrease was observed under low (0 and 4 mM) external K^+^ concentration (**Figure 6A**). At all external potassium concentrations, carvone reduced K_2P_3.1 currents (**Figures 6B, C**). Currents at 0 mM (no added potassium) external K^+^ were too low to allow further accurate analysis. When we analyzed the effect of carvone on each current component (VD or VI) at three external K^+^ concentrations, we found that the VD current was almost completely eliminated at all concentrations, while the VI current was only mildly affected (**Figures 6C–G**). At 60 mV, inhibition was reduced by high external K^+^ (**Figure 6C**).

## Discussion

In this study, we used an exogenous expression system to measure the impact of MTs on the activity of various human K_2P_ channels. MTs were found to affect various types of ion channels at high micromolar to millimolar concentrations (Lauritzen et al., 2005; Joca et al., 2012; Johnson et al., 2012; Brohawn et al., 2014; Pham et al., 2015; Bavi et al., 2016; Cabanos et al., 2017; Li Fraine et al., 2017), comparable to the concentrations that were found here to affect K_2P_ channels. Mechano-gated K_2P_4.1 and K_2P_10.1 were activated mainly by the cyclic aromatic phenolic MTs, carvacrol, and thymol, with the latter being the most effective. This is in accordance with our finding that the same MTs are the best activators of the other mechano-gated K_2P_ channel, K_2P_2.1 (Arazi et al., 2020). Unlike in K_2P_2.1 channels, the voltage dependency of the current did not change as a result of MT activation. Our findings suggest that for best activation of mechano-gated K_2P_ channels, terpenes should be moderately hydrophobic (XLogP3 ~ 3, as is the case for carvacrol and thymol) and to be able to penetrate, yet not become embedded in, the bilayer due to the presence of a small polar area. Also, a phenol moiety was necessary to obtain high channel-stimulating activity. We believe that such molecules are embedded into the outer leaflet of the bilayer and perturbed its structure and/or curvature. Less hydrophobic and more polar molecules (a polar area larger than 20 Å^2^ and XLogP value lower than 3) will stay in the polar area of the outer leaflet, while more hydrophobic molecules will sink deeper into the bilayer. We showed that for K_2P_2.1, the cytoplasmic carboxyl-terminal of the channel is needed for the activity of MTs (Arazi et al., 2020). It is yet to be determined whether this is the mechanism by which the other two mechano-gated K_2P_ channels are activated by MTs.

The alkaline-sensitive K_2P_5.1 and K_2P_17, but not K_2P_16.1, were also found to be activated by MTs. While K_2P_17.1 was only mildly activated (up to a fourfold increase in current, **Figure 3A**), K_2P_5.1 currents increased by up to 17-fold (0.3 mM, **Figure 2A**). Even at 60 µM carvacrol, K_2P_5.1 currents increased by 5.5-fold (**Figure 2C**). The selectivity of the MTs towards these channels was not the same as for mechano-gated channels. While carvacrol activated, to a degree, all channels, K_2P_5.1 was uniquely activated by cinnamaldehyde, but not by thymol, and K_2P_17.1 was uniquely activated by *β*-citronellol and 4MC (**Figure 3A**). K_2P_18.1 channels were activated by the same MTs, up to 5.3-fold (**Figure 3B**). Our findings indicate a certain degree of selectivity in the sensitivity of different K_2P_ channels to MTS, as some channels are activated by MTs that are inactive against other channels. The origin for this apparent selectivity is unclear since whether membrane-adhered hydrophobic molecules directly bind to channels or if they change membrane properties, causing each channel to react differently to those changes is an ongoing debate (Cristani et al., 2007; Ogawa et al., 2009; Lee, 2011; Nury et al., 2011; Epand et al., 2015; Sacchi et al., 2015). This dilemma applies not only to monoterpenes but also to other lipophilic molecules such as general anesthetics and alcohols (Howard et al., 2014), as well as for endocannabinoids (Oz, 2006) and steroids (Hill et al., 2015), all are allosteric modulators of several structurally different ion channels (Sanchez-Borzone et al., 2014; Oz et al., 2015; Ton et al., 2015). By changing the physicochemical properties of the surrounding membrane environment (Sanchez et al., 2004; Turina et al., 2006; Zunino et al., 2011; Reiner et al., 2013), and energetic requirements for gating-related conformational changes monoterpenes could affect ligand-gated ion channels (LGICs) (Fantini and Barrantes, 2009; Barrantes et al., 2010), and voltage-gated ion channel (VGICs) as reviewed by Oz at al. 2015 (Oz et al., 2015). On the other hand, evidence for direct binding of lipophilic monoterpenes, such as carvacrol and thymol, to specific amino acid residues in the transmembrane domain of Human 5-Hydroxytryptamine Type 3 (5-HT3Rs) were found (Lansdell et al., 2015). Similarly, the different potency of menthol stereoisomers (Walstab et al., 2014) on 5-HT3Rs or GABA(A) receptor (Corvalan et al., 2009) suggest also a degree of selectivity in monoterpenes action on ion channels.

For K_2P_2.1, it was shown that channel opening would result in a reduction in its voltage dependency (Bockenhauer et al., 2001; Lopes et al., 2005; Cohen et al., 2008). Schewe et al. displayed that, for all mechano-gated K_2P_ channels, activation causes a gating mode shift within the selectivity filter and that these channels can be converted into a “classical” leak mode when stimulated by arachidonic acid or PIP_2_ (Schewe et al., 2016). This phenomenon was observed in our experiments with K_2P_2.1, but not with K_2P_4.1 or K_2P_10.1 channels. Reduction in the percent of voltage-dependent current was observed with K_2P_5.1, but not with K_2P_17.1, while with K_2P_18.1, an increase in the voltage-dependent current was recorded (**Figure 3C**). The voltage-dependency of the K_2P_ channels’ current is directly related to their open probability. Since in our experiments, only a relative estimation of the open probability of the channel is measured, we believe that it is plausible that “leak-like” behavior of the channel is achieved only at high open probabilities and that under our experimental conditions, this was not always achieved.

The acid-sensitive TASK channels, K_2P_3.1 and K_2P_9.1, were affected differently by MTs: a. most tested MTs caused a decrease in TASK channel currents (**Figure 4A**); b. the current decrease rate was high (a few seconds), while the activation rate of other channels was at least an order of magnitude slower (**Figures 4B, C** and not shown); and c. unlike other tested K_2P_ channels, TASK channels were affected mostly by linear MTs (*β*-citronellol and geraniol, **Figure 4A**). These three observations suggest that TASK channels are affected by MTs by a different mechanism than the other tested channels. Thus, we further studied the characteristics of the current inhibition of TASK channels by MTs using K_2P_3.1 and carvone as a model.

It was clearly evident that carvone almost completely eliminated the voltage-gated portion of K_2P_3.1 currents (**Figures 5D–F**). Since voltage-dependent gating was shown to originate from the movement of three to four potassium ions into the high electric field of an inactive selectivity filter (Schewe et al., 2016) and since the stability of the selectivity filter was shown to be affected by the concentration of external potassium ions in potassium channels in general (Hille, 2001) and in K_2P_ channels in particular (Zilberberg et al., 2001), we looked at the influence of external potassium concentrations on K_2P_3.1 activity and sensitivity to carvone. External potassium ion levels had a clear effect on channel gating, as currents decreased dramatically at low external levels (**Figure 6A**). At all potassium levels, carvone reduced K_2P_3.1 current, while specifically targeting the voltage-dependent portion (**Figures 6C–G**). While the voltage-independent portion of the current behaved, as expected, like a potassium GHK-leak, the voltage-dependent portion displayed an outward rectification behavior that was partly dependent on potassium concentration (**Figure 6F**). We suggest that as external potassium ions stabilize the selectivity filter at its conductive state, they minimize the destabilizing structural changes caused by carvone. For this reason, we suggest that MTs might serve as a useful tool in studying the voltage-dependency of TASK channels as they specifically target the voltage-dependent portion of the current. Recently, it was reported that bupivacaine blocks TASK channels in a voltage-dependent manner by disrupting the K^+^-flux gating mechanism (Rinne et al., 2019), and that it is located laterally in the side fenestrations of K_2P_3.1 channels and interacts with residues of the pore helix, and the M2, M3, and M4 segments. It is conceivable that both bupivacaine and MTs bind to a similar binding site within the membrane and, thus, affect channels through a similar mechanism.

K_2P_ channels play a role in various physiological processes such as pain signaling (Li and Toyoda, 2015) heart function (Hancox et al., 2016), and more (Lesage and Barhanin, 2011; Bandulik et al., 2015; Renigunta et al., 2015; Riegelhaupt et al., 2018). For example, activation of mechano-gated K_2P_, as well as K_2P_18.1 channels, is expected to result in reduced pain sensation and neuroprotection. Terpenes have been proposed as analgesic agents (Khalilzadeh et al., 2016), as remedies for the treatment of pain and cardiovascular diseases (Magyar et al., 2004; Aydin et al., 2007; Menezes et al., 2010; Peixoto-Neves et al., 2010; Santos et al., 2011; Quintans-Junior et al., 2013; Quintans Jde et al., 2013; Guimaraes et al., 2014) and were shown to possess antitumor, local anesthetic, and anti-ischemic abilities (Koziol et al., 2014). Any of these activities of terpenes that stem from their activity on K_2P_ channels remains to be determined. Even though MTs are regularly consumed by people as food additives, due to their low concentration in food, they are unlikely to have any pharmacological effect. However, the extensive use of MTs in traditional medicine might raise the possibility of their beneficial pharmacological use when given in high concentrations.

## Data Availability Statement

All datasets generated for this study are included in the article/supplementary material.

## Ethics Statement

The animal study was reviewed and approved by Institutional Animal Care and Use Committee, Ben Gurion University. The project approval number is IL-61-09-2015.

## Author Contributions

Conception and design of the study: EA and NZ. Acquisition of data: EA and GB. Analysis and interpretation of data: EA. Writing the manuscript: EA and NZ.

## Funding

This work was supported by a grant from the Israel Science Foundation (1877/15) to NZ.

## Conflict of Interest

The authors declare that the research was conducted in the absence of any commercial or financial relationships that could be construed as a potential conflict of interest.

## Abbreviations

MTs, monoterpenes; K_2P_, two pore-domain potassium channels.
